# Supplementary material for: Phonon behavior in a random solid solution: a lattice dynamics study on the high-entropy alloy FeCoCrMnNi
Source: Nat Commun. 2022 Dec 6;13:7509. doi: 10.1038/s41467-022-35125-4 (PMC9726824; doi:10.1038/s41467-022-35125-4)
Supplement: Supplementary file 1 — Supplementary Information [file 41467_2022_35125_MOESM1_ESM.pdf]

Supplementary Material for "Phonon behavior in a random solid solution: A lattice dynamics study on the high-entropy alloy FeCoCrMnNi"

Shelby R. Turner,[1,2,3] Stéphane Pailhès,[3] Frédéric Bourdarot,[4] Jacques Ollivier,[1] Yvan Sidis,[5] John-Paul Castellan,[5,6] Jean-Marc Zanotti,[5] Quentin Berrod,[7] Florence Porcher,[5] Alexei Bosak,[8] Michael Feuerbacher,[9] Helmut Schober,[1] Marc de Boissieu,[2] and Valentina M. Giordano[3,*]

[1]*Institut Laue-Langevin, F-38042 Grenoble cedex, France*
[2]*Université Grenoble Alpes, CNRS, Grenoble-INP, SIMaP, F-38000 Grenoble, France*
[3]*Institute of Light and Matter, UMR5306 Université Lyon 1-CNRS, Université de Lyon, F-69622 Villeurbanne cedex, France*
[4]*Université Grenoble Alpes, CEA, IRIG, MEM, MDN, F-38000 Grenoble cedex, France*
[5]*Université Paris-Saclay, CNRS, CEA, Laboratoire Léon Brillouin, F-91191 Gif-sur-Yvette, France*
[6]*Institut für Festkörperphysik, Karlsruher Institut für Technologie, D-76021 Karlsruhe, Germany*
[7]*Université Grenoble Alpes, CEA, CNRS, IRIG-SyMMES, F-38000 Grenoble cedex, France*
[8]*European Synchrotron Radiation Facility, BP 220, F-38043 Grenoble cedex, France*
[9]*Peter Grünberg Institut PGI-5 and ER-C, FZ Jülich GmbH, D-52425 Jülich, Germany*
(Dated: November 17, 2022)

SUPPLEMENTARY NOTE 1: SAMPLE CHARACTERIZATION

Samples were investigated by Scanning Electron Microscopy (SEM) using a JEOL 840 microscope, equipped with an EDAX Energy-Dispersive X-ray (EDX) system, as seen in Fig. S1. The homogenized polycrystalline samples had an overall composition of $Fe_{19.89}Co_{20.97}Cr_{17.82}Mn_{19.54}Ni_{21.78}$ at.% with a homogeneous matrix. Small Cr-rich precipitates of about 1 μm in diameter were found with a volume fraction far below 1%. The overall composition of the Bridgman single crystal was determined to be $Fe_{20.00}Co_{19.64}Cr_{20.33}Mn_{20.10}Ni_{19.94}$ at.%. Optical microscopy of etched surfaces shows the presence of a dendritic structure on a 100 μm scale, which may be due to slight composition fluctuations. The single crystalline state and the primary orientation of the Bridgman crystal was determined using a Philips Micro X-ray Laue apparatus in back-reflection geometry. The produced crystal consisted of one dominant single grain with a volume of about 6 to 7 cm^3, and a number of minor secondary grains, which were cut off by spark erosion before further preparation. Oriented single crystalline samples were cut from the dominant single grain by spark erosion.

The polycrystalline sample was investigated by neutron diffraction at the Laboratoire Léon Brillouin (LLB) using the thermal-neutron two-axis powder diffractometer 3T-2@LLB with an incident wavelength $\lambda = 1.230$ Å at 300 K. As mentioned in the main text, a pattern matching with a LeBail fit was applied to refine the Bragg peaks according to an FCC structure within the $Fm\bar{3}m$ space group. In this type of refinement, only the geometrical parameters (sample cell parameters, peak width parameters /experimental wavelength, instrumental peak shape and peak width function including asymmetry, $2\theta_0$ correction) are accounted for, and peak intensities are refined directly against experimental ones, without accounting for the crystal structure.

Diffraction data were collected at the ID28@ESRF diffractometer at a wavelength of $\lambda = 0.697$ Å. They are reported in Figure S2. There is a clear anisotropic distribution of diffuse scattering located around the Bragg peaks. The usual thermal diffuse scattering (TDS) leads to an anisotropy characterized by a stronger diffuse scattering in the transverse than in the longitudinal direction. Looking at the diffuse scattering around the 200 Bragg peaks, it is clear that the opposite is true: there is a minimum of diffuse scattering in the transverse direction.

Such a signal was confirmed by measuring the purely elastic signal in transverse and longitudinal geometries during phonon measurements by IXS, shown in Fig. S3. Here we report the elastic signal from scans made (a) along 0 0 2+x and x x 2 lines and (b) along 2+x 2+x 0 and 2 2 x lines. The plots clearly show a significantly larger intensity in the LA directions that, in both cases, follows $1/q^2$ decay fit. This is a clear signature of the so-called Huang scattering. It is beyond the scope of this paper to provide a full quantitative analysis of it, but this illustrates the presence of strain induced by the distribution of atomic size differences of the five elements.

Finally, in a preliminary neutron diffuse scattering experiment at the D7 beamline of ILL, we could observe weak signatures of a possible short range ordering on a 10 Å lengthscale. However, this result needs to be confirmed by further studies.

SUPPLEMENTARY NOTE 2: METHODS: INELASTIC SCATTERING MEASUREMENTS

Taking first the example of a neutron scattering experiment, the measured quantity is the double differential cross-section, $\frac{d^2\sigma^n}{d\Omega_f dE_f}$,which is the number of neutrons out of incident neutrons that are scattered from a sample and into solid angle element Ω_f in a given energy

* To whom correspondence should be addressed: valentina.giordano@univ-lyon1.fr

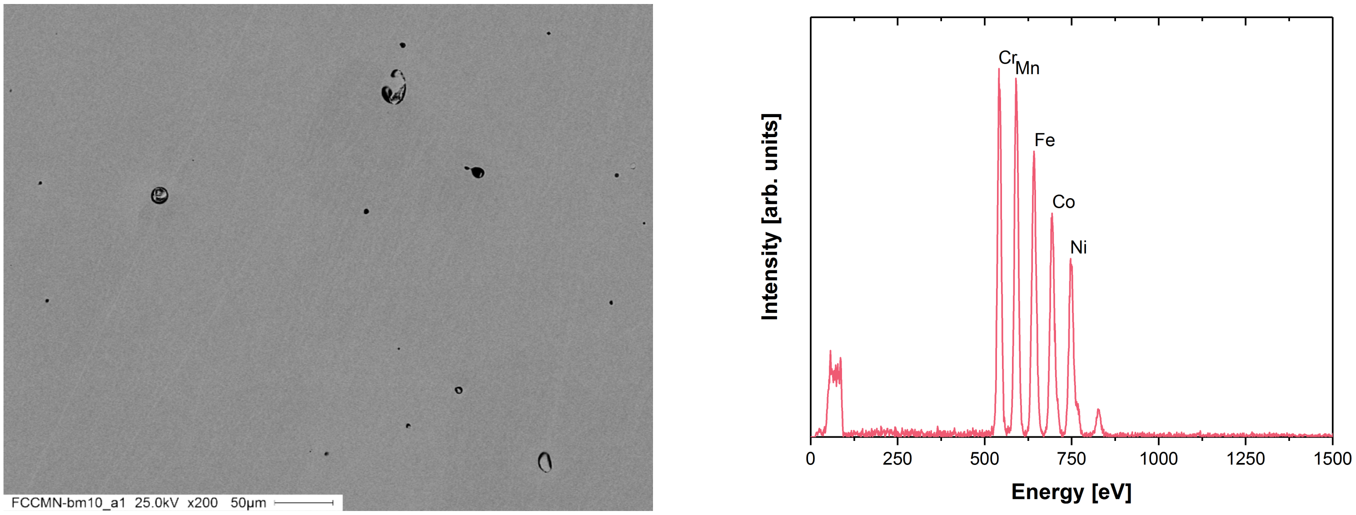

FIG. S1. The left image shows a backscattered-electron image of a polished surface of the FeCoCrMnNi crystal. Apart from several visible pores (black spots), the gray contrast is homogeneous. This means that the composition is homogeneous. The right image is an EDAX Energy-Dispersive X-ray (EDX) spectrum of an area corresponding to about the same size as the lefthand image.

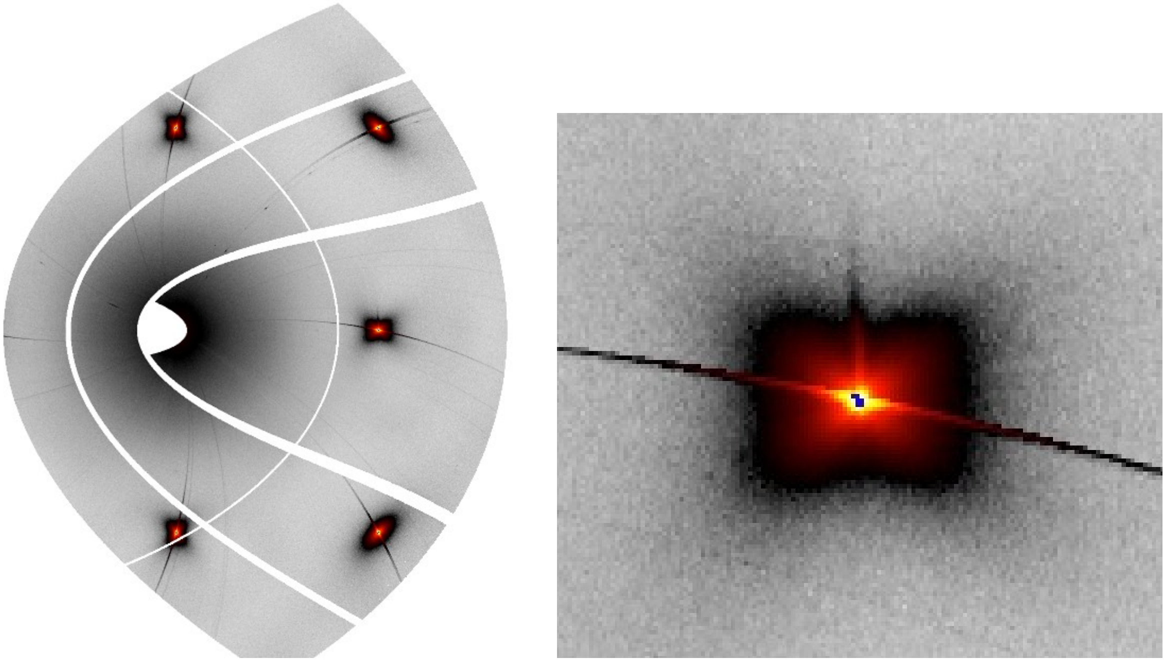

FIG. S2. Diffuse X-ray scattering of the FeCoCrMnNi single crystal sample measured in the HK0 Plane at 90 K (left) and around the 200 Bragg in the HK0 plane (right). There is a clear anisotropy with a minimum of diffuse scattering intensity along the 2 x 0 line as compared to the 2+x 0 0 line. This is opposite to the usual thermal diffuse scattering, and is typical for Huang scattering. Data are represented in a mixed lin-log scale as implemented in Albula by Dectris.

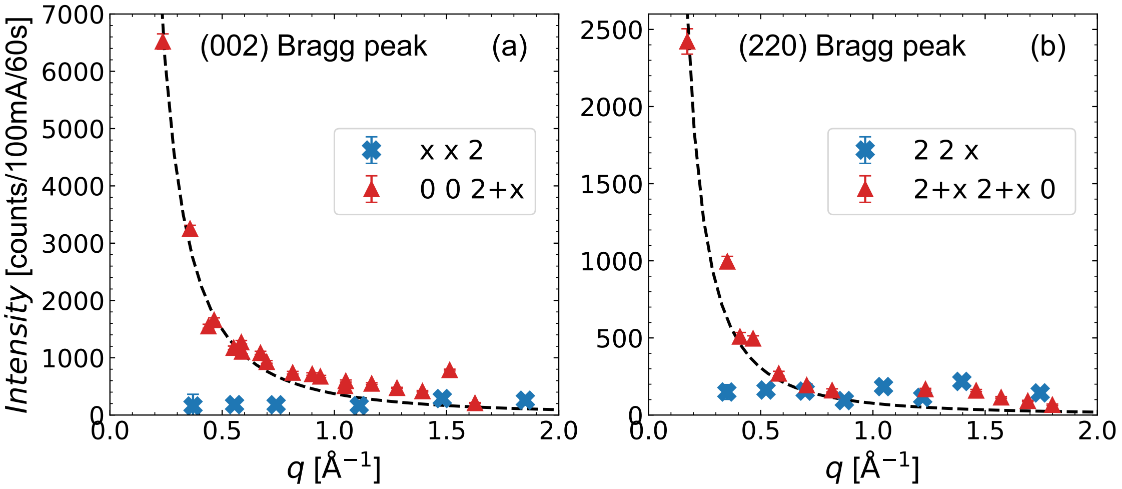

FIG. S3. Anisotropy of the elastic signal measured around the (002) and (220) Bragg peaks. In (a), elastic signal measured around the (002) Bragg peak for 0 0 2+x (longitudinal direction, red triangle) and x x 2 lines (transverse direction, blue crosses). The black dashed line is a $1/q^2$ decay fit. In (b), elastic diffuse scattering measured around the (220) Bragg for 2+x 2+x 0 (longitudinal direction, red triangle) and 2 2 x lines (transverse direction, blue crosses), with a similar $1/q^2$ decay fit. All intensities have been normalized based on efficiencies of the analyzers in order to compare scans across different ID28@ESRF analyzers.

range E_f. This has coherent and incoherent scattering components [1] and for incoming wave-vector k_i and outgoing wave-vector k_f, it is dependent upon the response function, $S(\mathbf{Q},\omega)$:

$$\begin{aligned}\frac{d^2\sigma^{\rm n}}{d\Omega_f dE_f} &= \left.\frac{d^2\sigma^{\rm n}}{d\Omega_f dE_f}\right|_{\rm coh} + \left.\frac{d^2\sigma^{\rm n}}{d\Omega_f dE_f}\right|_{\rm inc} \\ &= \frac{k_f}{k_i} S(\mathbf{Q},\omega)\end{aligned} \tag{S1}$$

$\mathbf{Q} = \mathbf{G} + \mathbf{q}$ being the position in reciprocal space we are measuring according to reciprocal lattice vector $\mathbf{G}$ and small displacement $\mathbf{q}$.

Coherent scattering and incoherent scattering, also called self-scattering, explain correlated atomic movements and individual atomic movements, respectively. When conducting a neutron or x-ray scattering experiment it is important to have an idea of the amount of incoherent and coherent scattering you can expect from a given sample. Continuing with the example of a neutron scattering experiment, we rely on the coherent neutron scattering length, b, of each element. The coherent, $\sigma^{\rm n}_{\rm coh}$, and incoherent, $\sigma^{\rm n}_{\rm inc}$, scattering cross-sections are defined as the following [2, 3]:

$$\begin{aligned}\sigma^{\rm n}_{\rm coh} &= 4\pi\langle b\rangle^2 \\ \sigma^{\rm n}_{\rm inc} &= 4\pi(\langle b^2\rangle - \langle b\rangle^2)\end{aligned} \tag{S2}$$

The neutron scattering lengths and scattering cross-sections for the elements of FeCoCrMnNi are given in Table S1. For FeCoCrMnNi, $\sigma^{\rm n}_{\rm coh} = 2.465$ barns/atom, and the total (spin, isotopic, and Laue scattering in the case of FeCoCrMnNi) $\sigma^{\rm n}_{\rm inc} = 2.526 + 3.280 = 5.806$ barns/atom, meaning that we expect 2.3× as much incoherent scattering as coherent scattering, and that incoherent scattering makes up 70% of the total neutron scattering cross-section of the material. In our experiment, assuming that the phonon modes represent the amount of coherent inelastic neutron scattering for a given $\mathbf{Q}$ scan and that the observed broad band accounts for the incoherent neutron scattering, we find this latter to be ∼2.8 times the coherent phonon signal for TA^{010}_{100} and ∼2.1 times for $\mathrm{TA}^{110}_{\bar{1}10}$, in very good agreement with the expected incoherent-to-coherent ratio.

TABLE S1. The neutron scattering lengths, b, and neutron coherent and incoherent scattering cross-sections, $\sigma^{\rm n}_{\rm coh}$ and $\sigma^{\rm n}_{\rm inc}$, are provided for the elements present in FeCoCrMnNi. Scattering cross sections are given in barns (1 barn = 10^{-24} cm^2) and neutron scattering lengths are given in femtometers (1 fm = 10^{-13} cm) [2].

	**b**	$\boldsymbol{\sigma^{\rm n}_{\rm coh}}$	$\boldsymbol{\sigma^{\rm n}_{\rm inc}}$
**Fe**	9.45	11.22	0.4
**Co**	2.49	0.779	4.8
**Cr**	3.635	1.66	1.83
**Mn**	-3.73	1.75	0.4
**Ni**	10.3	13.3	5.2

In addition, the measured incoherent component of the response function, $S_{\rm inc}(\mathbf{Q},\omega)$, is written as

$$S_{\rm inc}(\mathbf{Q},\omega) = Q^2 g(\omega)(n(\omega)+1)/\omega, \tag{S3}$$

where $g(\omega)$ is the generalized vibrational density of states (GVDOS), and $n(\omega)$ is the Bose occupation factor.

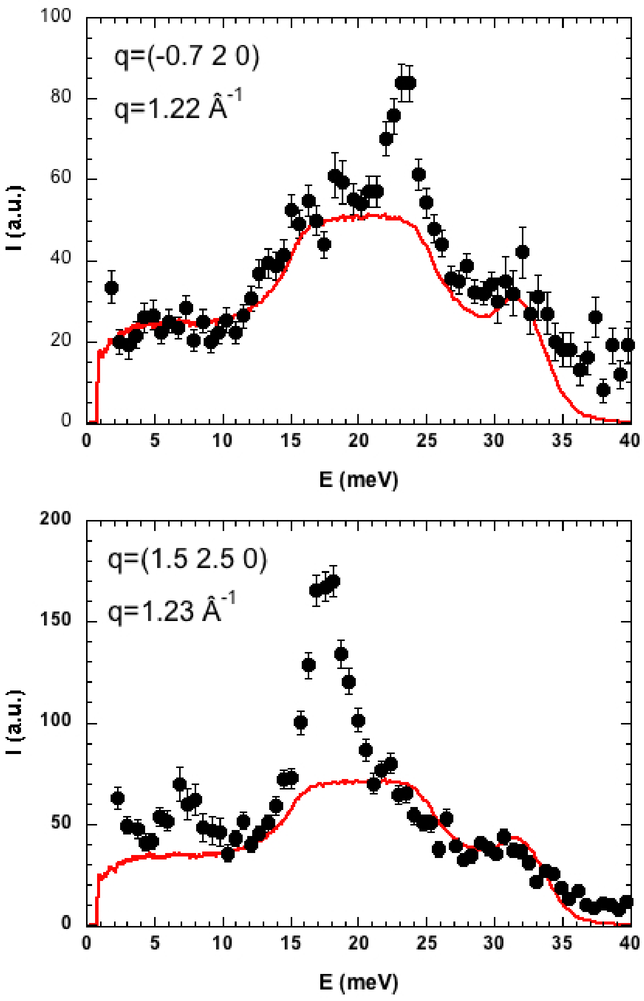

FIG. S4. Comparison between the measured inelastic neutron scattering intensity for two transverse acoustic (TA) excitation (solid black circles) and the simulated incoherent contribution (solid red line, see text). The top panel is for the TA phonon (-0.7 2 0) and the bottom panel is for the TA phonon (1.5 2.5 0).

Using the measured GVDOS from the main text and applying the $(n(\omega)+1)/\omega$ rescaling, the incoherent signal has been simulated. It is shown as a red line in Fig. S4 where it is compared to the data for two transverse excitations measured at high q positions. The incoherent signal is clearly dominating, noticing, in particular, the constant intensity observed and simulated at low q. To be rigorous, however, a multiphonon contribution should be added at high energy.

The coherent and incoherent X-ray scattering can as well be calculated by starting again from the double differential cross-section for a scattering event, such as the one given for neutrons in eq. S1. In this case we are dependent on the radius of the electron, r_e, and the polarization of the incoming (outgoing) photon, $\hat{\epsilon}_i$ ($\hat{\epsilon}_f$) [4].

$$\frac{d^2\sigma^{\mathrm{x}}}{d\Omega_f dE_f} = r_e^2(\hat{\epsilon}_f \cdot \hat{\epsilon}_i)^2 \frac{k_f}{k_i} S(\mathbf{Q},\omega) \tag{S4}$$

Then the coherent and incoherent X-ray scattering cross-sections, $\sigma^{\mathrm{x}}_{\mathrm{coh}}$ and $\sigma^{\mathrm{x}}_{\mathrm{inc}}$, respectively, depend upon the atomic form factors, f, and are written as follows [5]:

$$\begin{aligned} \sigma^{\mathrm{x}}_{\mathrm{coh}} &= \langle f \rangle^2 \\ \sigma^{\mathrm{x}}_{\mathrm{inc}} &= \langle f^2 \rangle - \langle f \rangle^2 \end{aligned} \tag{S5}$$

As a first approximation we will take the values at $\mathbf{q} = 0$, i.e. $\mathbf{Q} = \mathbf{G}$, meaning that the atomic form factor for each element is defined as $f = Z$, where Z is the atomic number, directly from the Periodic Table of Elements. Therefore, $\sigma^{\mathrm{x}}_{\mathrm{coh}} = 676$, and $\sigma^{\mathrm{x}}_{\mathrm{inc}} = 2$, confirming that incoherent X-ray scattering for FeCoCrMnNi is minimal in comparison to the coherent component.

A. Time-of-Flight Spectroscopy

Time-of-Flight (TOF) spectroscopy was used to measure the neutron-weighted generalized vibrational density of states (GVDOS) reported in Fig. 2 from the main text. A single crystal sample of FeCoCrMnNi (cylinder-shaped with a height of 1 cm and diameter of 0.8 cm) was measured on the cold-neutron TOF spectrometer IN5@ILL with an incident wavelength of $\lambda = 3.2$ Å at room temperature with a cryostat environment. The sample was rotated 0.5° after each scan, resulting in a total Ω range of 43°.

A polycrystalline sample (2x1x1 cm^3, 11.5 g) was taken to the cold-neutron TOF spectrometer IN6@ILL and measured with a Be filter and $\lambda = 5.1$ Å at 100, 200, and 300 K, also using a standard cryostat. Empty can measurements, or measurements that are made in the same sample environment but without the sample in order to account for non-sample-related scattering captured at the detectors, were taken at all three temperatures and subtracted from the sample data. Fig. S5 reports an example of raw spectrum collected at IN6 at 300 K, in time of flight units, before and after the emoty cell subtraction, the empty cell being also reported in the figure.

Both IN5@ILL and IN6@ILL GVDOS plots were produced using the MUPHOCOR (MUlti-PHOnon CORrection) program [6] for LAMP [7], in which the expected neutron scattering cross section and atomic mass of FeCoCrMnNi were used as starting parameters for a self-consistent multi-phonon scattering correction of the data. Fig. S6 reports an example of the GVDOS collected at 300 K at IN5, before and after the multiphonon correction. The multiphonon contribution calculated by the self-consistent iterative procedure is reported as a shaded area.

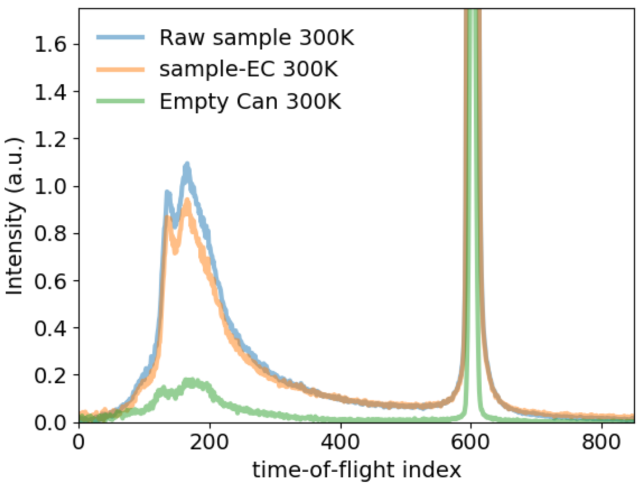

FIG. S5. Example of raw data collected at IN6 on the polycrystalline sample at 300 K in time of flight units, before (blue) and after (orange) empty cell subtraction. The empty cell is also reported as a green line.

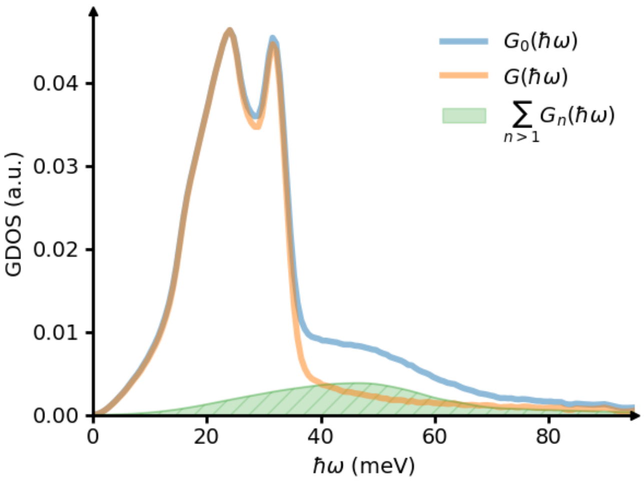

FIG. S6. Example of the GVDOS collected at 300 K at IN5, before (blue, $G_0(\hbar\omega)$) and after (orange, $G(\hbar\omega)$) the multiphonon correction. The multiphonon contribution is reported as a green shaded area ($\sum_{n>1} G_n(\hbar\omega)$).

B. Triple-Axis Spectroscopy

The transverse acoustic (TA) mode propagating along [$\bar{1}$00], polarized along [010] ($TA^{010}_{\bar{1}00}$), and the TA mode propagating along [$\bar{1}$10], polarized along [110] ($TA^{110}_{\bar{1}10}$) shown in Fig. 3(c,d) of the main text were taken from Triple-Axis Spectroscopy (TAS) measurements on the thermal-neutron TAS instrument 1T-1@LLB. A fixed k_f = 2.662 Å^{-1} (λ = 2.36 Å) and cryostat were used in order to take constant **Q** scans at 3, 100, and 300 K.

The TA mode and the textured band were fit using the program 'AFITV,' an analysis tool developed by B. Hennion and P. Bourges at Laboratoire Léon Brillouin [8]. It takes into account instrumental resolution by using instrumental parameters as well as sample characteristics, in order to model the shape and size of the experimental resolution. Indeed, the instrumental resolution is energy and q dependent and is determined from the experimental setup, the phonon group velocity, and the sample mosaicity, which is a measure of the quality of a single crystal. The mosaicity measures the angular distribution of a given direction in the reciprocal space, deteriorating the resolution in q and thus in energy. The mosaicity of our single crystal was about 1°, which results in a total energy resolution between 2.2 and 1.3 meV depending on q.

AFITV makes the convolution of the scattering function and instrumental resolution function [9], therefore allowing us to decouple the intrinsic phonon measurement from the distortion caused by the spectrometer. More details on the use of this software are discussed in Appendix III.

Unfortunately, the presence of the incoherent scattering made the fitting procedure particularly challenging, so that, even if at high energy the phonon linewidth was not resolution limited, we could not reliably extract it because of the merging of the phonon with the incoherent intensity.

C. Inelastic X-ray Scattering

Inelastic X-ray scattering was used to measure the longitudinal acoustic (LA) modes propagating along the [001] and [110] directions (LA_{001} and LA_{110}) and and the transverse acoustic (TA) modes propagating along [00$\bar{1}$], polarized along [110] ($TA^{110}_{00\bar{1}}$) and propagating along [110], polarized along [001] (TA^{001}_{110}) reported in Fig. 3 of the main text. Measurements were made at the IXS ID28@ESRF beamline. The [999] reflection of the silicon monochromator was used, resulting in an incoming X-ray energy of 17.794 keV (or a wavelength of 0.697 Å) and an energy resolution of 2.8 meV. The Joule-Thomson displex was incorporated for temperature measurements at 15, 100, and 300 K.

The ID28@ESRF beamline has the advantage of being able to measure 9 points in reciprocal space simultaneously, due to the 9 analyzers mounted at fixed 2θ angular distances of 1.54° from each other. The plots in Fig. 3(a,b) from the main text and Figs. S21-S23 represent scans from all 9 of these analyzers, and therefore the intensities should not be compared directly, due to the fact that each analyzer has its own efficiency as compared to the others. All analyzers were receiving the scattered intensity through rectangular slits, with horizontal and vertical opening of 20 mm and 55 mm respectively. The horizontal opening fixes the q resolution which is Δq = 0.026 Å^{-1}. This q resolution induces an additional energy broadening as it couples to the slope of the acoustic dispersion: $\Delta E_q = v_g \Delta q$, where v_g is the

group velocity. As such it plays a major role at low q, before the bending of the acoustic dispersion. As for the spectrometer energy resolution, this has been measured for each of the 9 analyzers by measuring the elastic scattering of a plexiglass sample, cooled to 14.5 K, at q values close to the maximum of the static structure factor, within the energy range ± 40 meV. Finally, the crystal used for these measurements had a very small mosaicity of only 0.1°, leading to a mosaicity contribution to the effective resolution of only 0.1 meV.

In order to extract the intrinsic phonon linewidths shown in Figs. 6,7,8 from the main text, both energy and q resolution (ΔE, Δq) have been taken into account. For this, the q distribution over the illuminated area of the analyzer has been calculated and the experimental spectrum has been fit with a superposition of phonon modes for all q's within this distribution, then convoluted with the energy instrumental resolution.

SUPPLEMENTARY NOTE 3: ACOUSTIC NATURE OF THE PHONON MODES

In both INS and IXS experiments we have fit our data using a delta function for the elastic line, a damped harmonic oscillator for the phonon mode, and, in the INS case, a two-part Gaussian distribution for the broad textured band. The fit was performed using AFITV for INS data and a home-made Matlab program for IXS. In both programs the theoretical model was convoluted with the instrumental resolution function prior to fitting the experimental data, allowing for the extraction of the intrinsic phonon properties (position, intensity and linewidth). Fitting the different components of the scans in this manner allows us to account for all of the intensity shown in the scans.

The result of this method of analysis is that we can track the changes in the normalized intensity of $S(\mathbf{Q}, \omega, T)$, from eq. S1, of the phonon mode to determine its acoustic character. It can be shown that for an acoustic phonon, the normalized dynamic structure factor, $\mathrm{DSF}_{i,\mathbf{q}}(\mathbf{Q}, \omega, T)$, is constant, where the integral is taken over the measured phonon peak [3, 10–13]. The response function, $S(\mathbf{Q}, \omega, T)$, defined in Appendix II, depends on the thermal occupation factor, $n(\omega_{i,\mathbf{q}})$, the polarization of the phonon mode, $\xi_{i,\mathbf{q}}$, and the Bragg peak structure factor, F_B. This equation works in the long-wavelength limit, $|\mathbf{q}| \ll |\mathbf{Q}|$, and given that $\hbar\omega \ll k_B T$.

$$\mathrm{DSF}_{i,\mathbf{q}}(\mathbf{Q}, \omega, T) = \frac{\omega_i(\mathbf{q})}{Q^2 \cdot n(\omega_{i,\mathbf{q}})} \int S_{i,\mathbf{q}}(\mathbf{Q}, \omega, T) d\omega,$$

$$\text{where} \int S_{i,\mathbf{q}}(\mathbf{Q}, \omega, T) d\omega \approx (\mathbf{Q} \cdot \xi_{i,\mathbf{q}})^2 |F_\mathrm{B}|^2 \frac{n(\omega_{i,\mathbf{q}})}{\omega_i(\mathbf{q})} \tag{S6}$$

In Fig. S7 we report the normalized intensity of the phonon mode across the Brillouin zone in each direction, as obtained with the fit of INS data. In order to simplify the analysis, we have imposed a constant energy

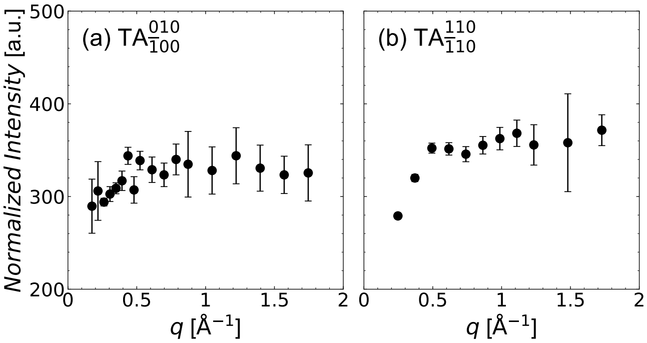

FIG. S7. Normalized intensity (see eq. S6) of the two phonon modes (see figure labels) measured by constant-$\mathbf{Q}$ inelastic neutron scattering scans on 1T@LLB at 300 K.

position and normalized intensity for the main features of the broad band across the Brillouin zone for a given direction. This is justified by the fact that the broad band looks q independent, as shown in Fig. S10. Above 0.4 Å^{-1}, the normalized intensity of the phonon modes remain constant within the errorbars up to the Brillouin zone border, confirming the acoustic nature of the transverse modes throughout the Brillouin zone. A slight decrease at smaller q, below 0.4 Å^{-1}, in both directions, is in fact due to a strong entanglement of the intensities of TA and broad band with the intense elastic line, making these values more uncertain. The constant character of the phonon normalized intensity supports the conclusion that there is no interference of the phonon with the broad band. This is different from the case of other complex crystalline systems in which an intensity transfer is observed between acoustic modes and low-lying optical modes [14–19].

In Fig. S8 we report the normalized intensity of the four polarizations measured by IXS. All intensities have been normalized by the analyzer efficiencies in order to compare scans across different analyzers. If along the [110] direction in (c,d) the normalized intensity remains quite constant, along the [001] direction in (a,b) this is not so evident. Specifically, a deviation from constant clearly appears in the longitudinal polarization in (a) at ∼ 0.8-1 Å^{-1}, which coincides with the third regime of the longitudinal attenuation, described in Fig. 6(a) of the main text. Still, we can conclude that the acoustic character is conserved up to at least 1 Å^{-1}.

SUPPLEMENTARY NOTE 4: HEA DENSITY OF STATES COMPARISON

In Fig. S9, Generalized Vibrational Density of States (GVDOS) of equiatomic FeCoCrNi (FCCN) [20] at 300 K is plotted against the GVDOS of FeCoCrMnNi, replotted from Fig. 2(b) from the main text. Both measurements have been made by INS, and show close matching, including in the low energy acoustic region between 0-10

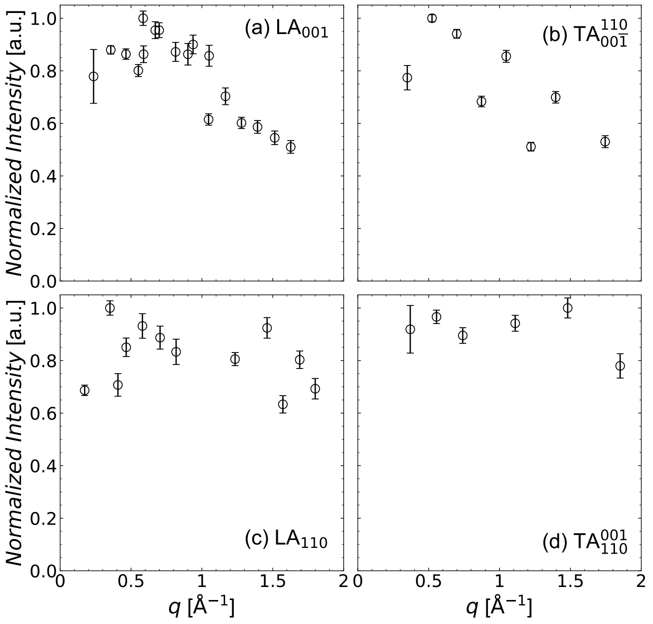

FIG. S8. Normalized intensity (see eq. S6) of the four phonon modes (see figure labels) measured by constant-$\mathbf{Q}$ inelastic X-ray scattering scans on ID28@ESRF at 300 K. All intensities have been normalized based on efficiencies of the analyzers in order to compare scans across different analyzers.

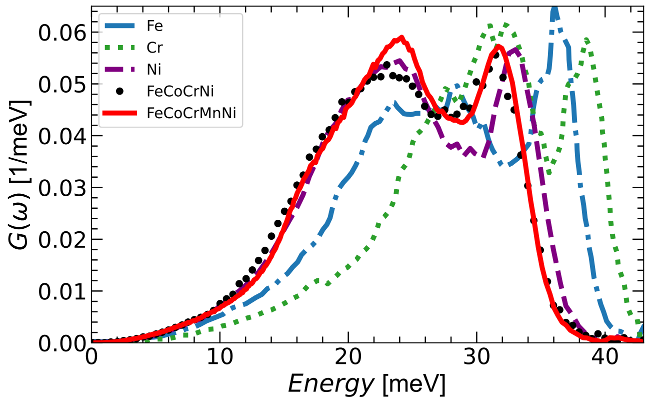

FIG. S9. The Generalized Vibrational Density of States of FeCoCrMnNi (solid red line), shown first in Fig. 2(b) from the main text, is compared to the GVDOS of FeCoCrNi (black circle markers), measured by Lucas *et al.* with Inelastic Neutron Scattering, and to several single element GVDOS, including Fe [21] (blue dash-dotted line), Ni [22] (purple dashed line), and Cr [21] (green square markers).

meV. These are additionally compared to elemental GVDOS measurements of Fe [21], Ni [22], and Cr [21]. The GVDOS of FeCoCrMnNi and FeCoCrNi appear to most closely match that of Ni.

SUPPLEMENTARY NOTE 5: INELASTIC SCATTERING ENERGY SCANS

All scans used to create Fig. 5 of the main text that were not already shown in Fig. 3 of the main text have been included in the following section. The scans taken during the neutron triple-axis spectroscopy experiment at 300 K are shown in Fig. S10. For the inelastic neutron scattering experiment, Figs. S11-S12 depict scans at 100 and 3 K from the $TA^{010}_{\bar{1}00}$ dispersion, respectively, and similarly for Figs. S13-S14 and the $TA^{110}_{\bar{1}10}$ dispersion.

For the inelastic X-ray scattering measurements, Figs. S15-S17 show scans from the LA_{001} polarization at 300, 100, and 15 K, respectively, and similarly for the $TA^{110}_{00\bar{1}}$ polarization in Figs. S18-S20. Scans for the LA_{110} polarization, taken at 300 K, are plotted in Fig. S21. Finally, the TA^{001}_{110} polarization measurements at 300 and 15 K are represented in Figs. S22 and S23, respectively.

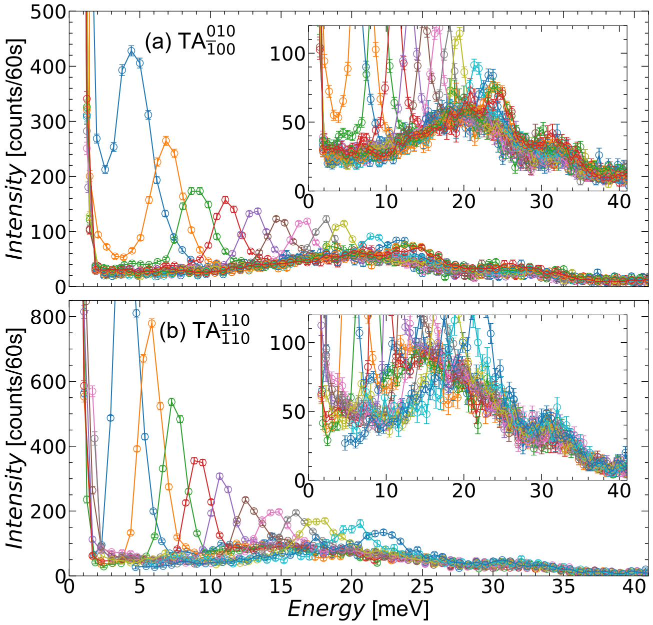

FIG. S10. Plots of the (a) $TA^{010}_{\bar{1}00}$ and (b) $TA^{110}_{\bar{1}10}$ phonon dispersions at 300 K, measured by inelastic neutron scattering, which emphasize the incoherent scattering seen in all neutron scans. The broad textured band persists through the entire Brillouin zone in both polarizations. In (a), scans cover $q = 0.1 - 0.6$ r.l.u. with a step size of 0.05 r.l.u., and $q = 0.6 - 1.0$ r.l.u. with a step size of 0.1 r.l.u. In (b), scans cover $q = 0.1 - 0.5$ r.l.u. with a step size of 0.05 r.l.u., and $q = 0.5 - 0.7$ r.l.u. with a step size of 0.1 r.l.u.

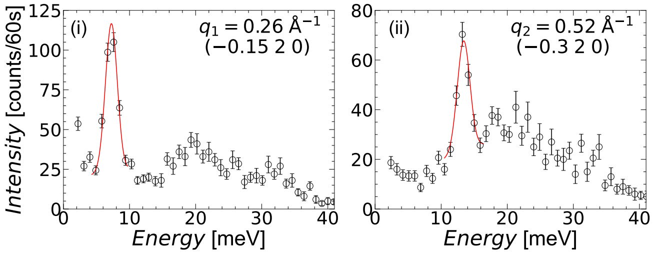

FIG. S11. Energy scans at 100 K of the transverse acoustic (TA) mode propagating along the $[\bar{1}00]$, polarized along [010] ($TA^{010}_{\bar{1}00}$), taken near the (020) Bragg peak. Experiments were made at 1T@LLB, using a fixed k_f of 2.662 Å^{-1}.

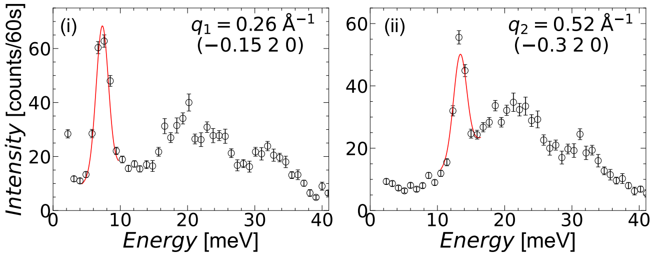

FIG. S12. Energy scans at 3 K of the transverse acoustic (TA) mode propagating along the $[\bar{1}00]$, polarized along [010] ($TA^{010}_{\bar{1}00}$), taken near the (020) Bragg peak. Experiments were made at 1T@LLB, using a fixed k_f of 2.662 Å^{-1}

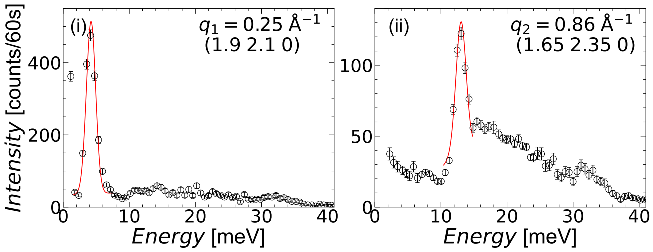

FIG. S13. Energy scans at 100 K of the transverse acoustic (TA) mode propagating along the $[\bar{1}10]$, polarized along [110] ($TA^{110}_{\bar{1}10}$), taken near the (220) Bragg peak. Experiments were made at 1T@LLB, using a fixed k_f of 2.662 Å^{-1}

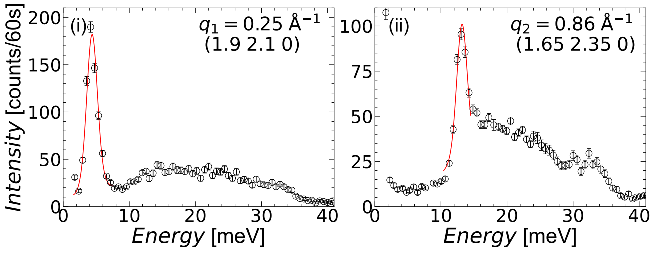

FIG. S14. Energy scans at 3 K of the transverse acoustic (TA) mode propagating along the $[\bar{1}10]$, polarized along [110] ($TA^{110}_{\bar{1}10}$), taken near the (220) Bragg peak. Experiments were made at 1T@LLB, using a fixed k_f of 2.662 Å^{-1}

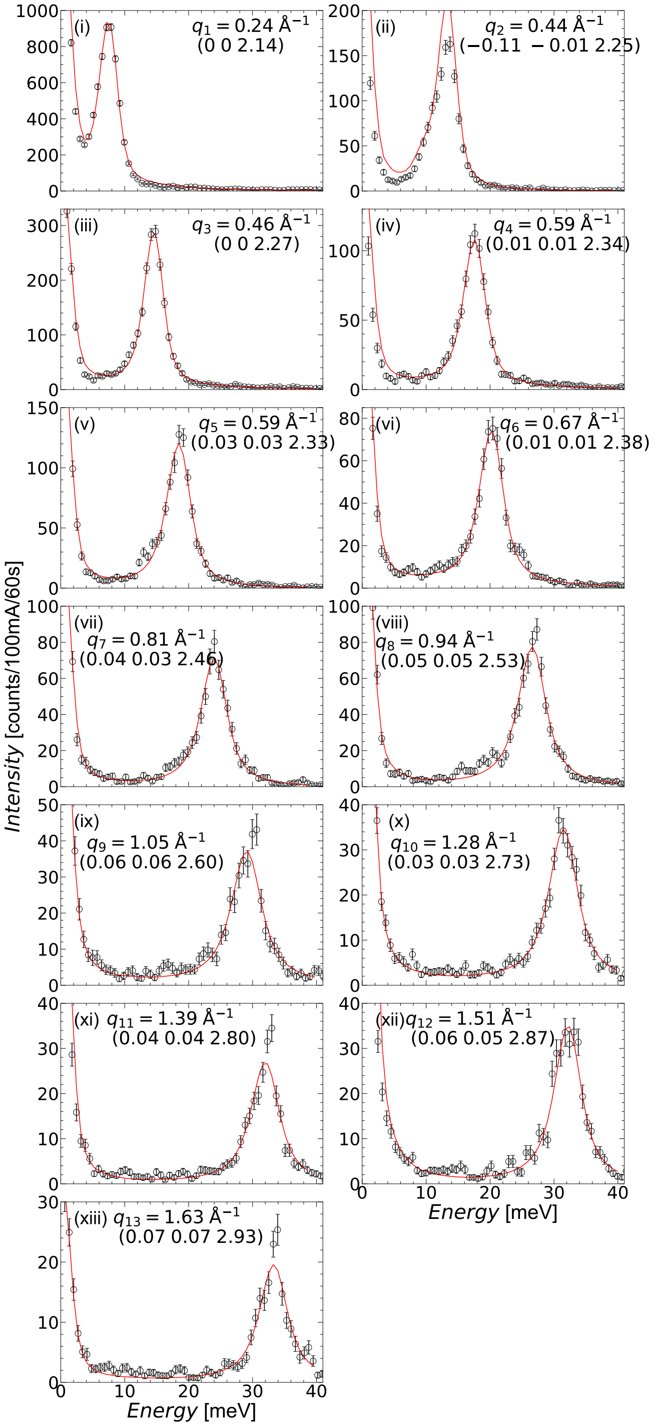

FIG. S15. Energy scans at 300 K of the longitudinal acoustic (LA) mode propagating along the [001] direction (LA_{001}), taken near the (002) Bragg peak. Experiments were made at the ID28@ESRF beamline, using the [999] reflection of the silicon monochromator.

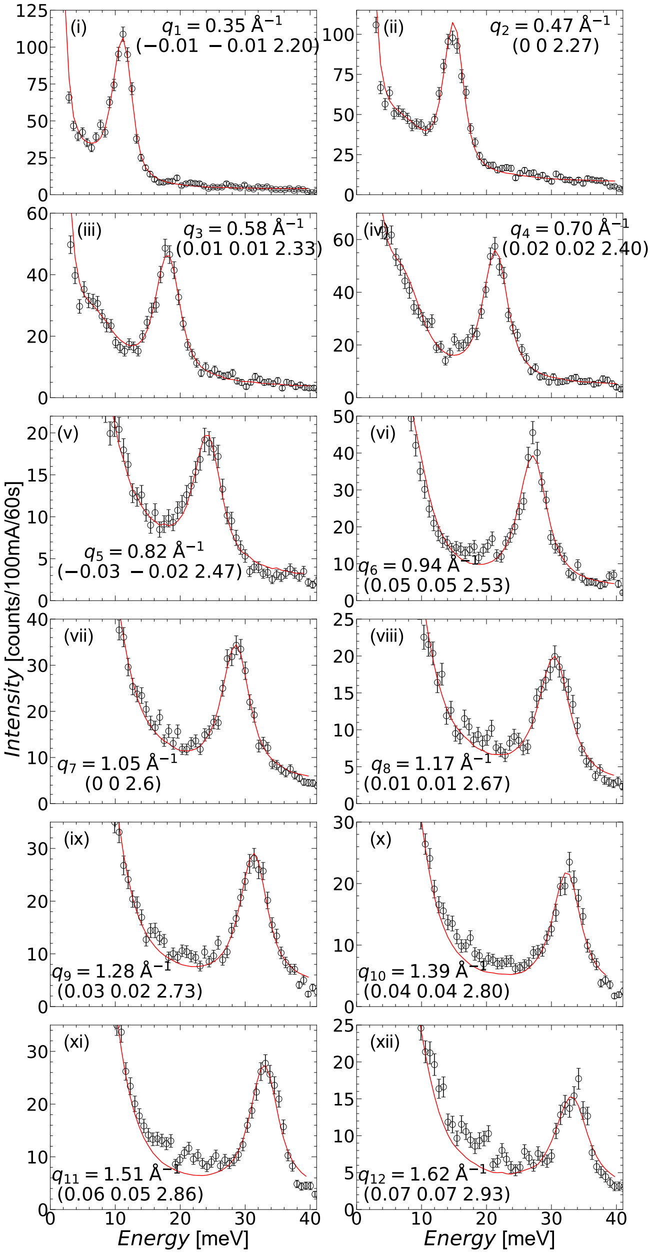

FIG. S16. Energy scans at 100 K of the longitudinal acoustic (LA) mode propagating along the [001] direction (LA_{001}), taken near the (002) Bragg peak. Experiments were made at the ID28@ESRF beamline, using the [999] reflection of the silicon monochromator.

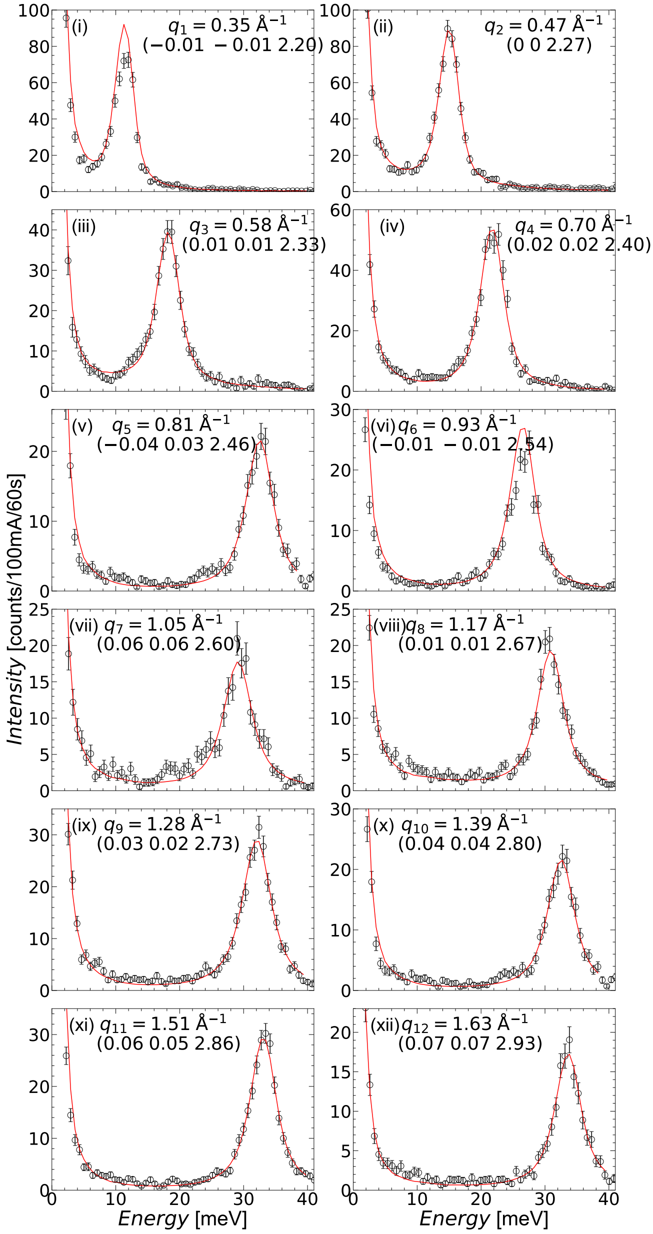

FIG. S17. Energy scans at 15 K of the longitudinal acoustic (LA) mode propagating along the [001] direction (LA_{001}), taken near the (002) Bragg peak. Experiments were made at the ID28@ESRF beamline, using the [999] reflection of the silicon monochromator.

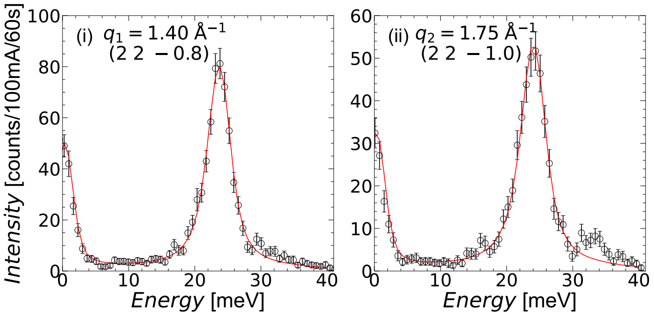

FIG. S18. Energy scans at 300 K of the transverse acoustic (TA) mode propagating along [00$\bar{1}$], polarized along [110] ($TA_{00\bar{1}}^{110}$), taken near the (220) Bragg peak. Experiments were made at the ID28@ESRF beamline, using the [999] reflection of the silicon monochromator.

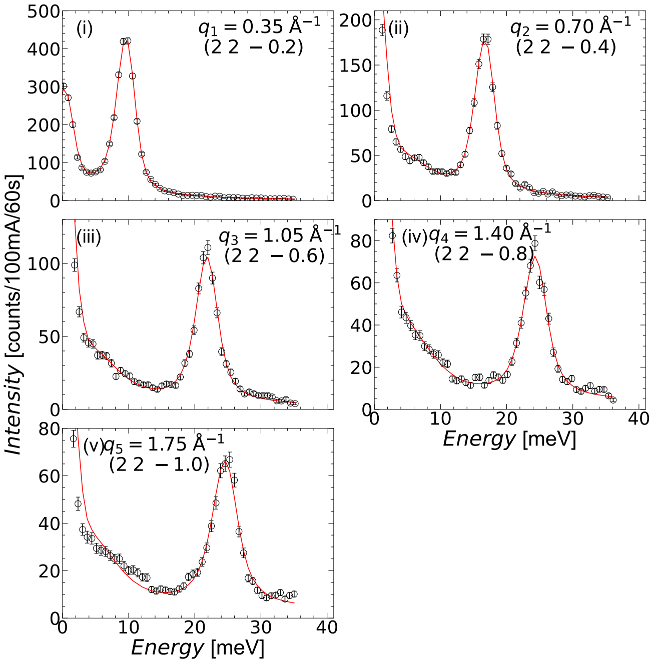

FIG. S19. Energy scans at 100 K of the transverse acoustic (TA) mode propagating along [00$\bar{1}$], polarized along [110] ($TA_{00\bar{1}}^{110}$), taken near the (220) Bragg peak. Experiments were made at the ID28@ESRF beamline, using the [999] reflection of the silicon monochromator.

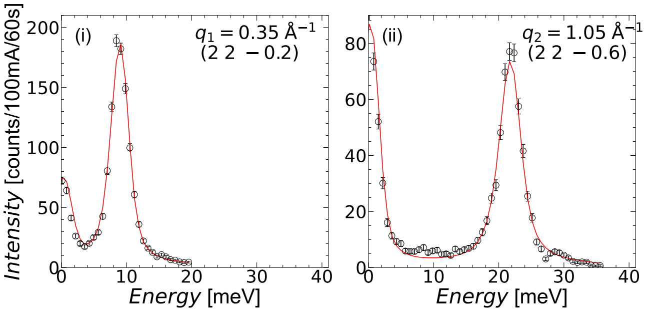

FIG. S20. Energy scans at 15 K of the transverse acoustic (TA) mode propagating along [00$\bar{1}$], polarized along [110] ($TA_{00\bar{1}}^{110}$), taken near the (220) Bragg peak. Experiments were made at the ID28@ESRF beamline, using the [999] reflection of the silicon monochromator.

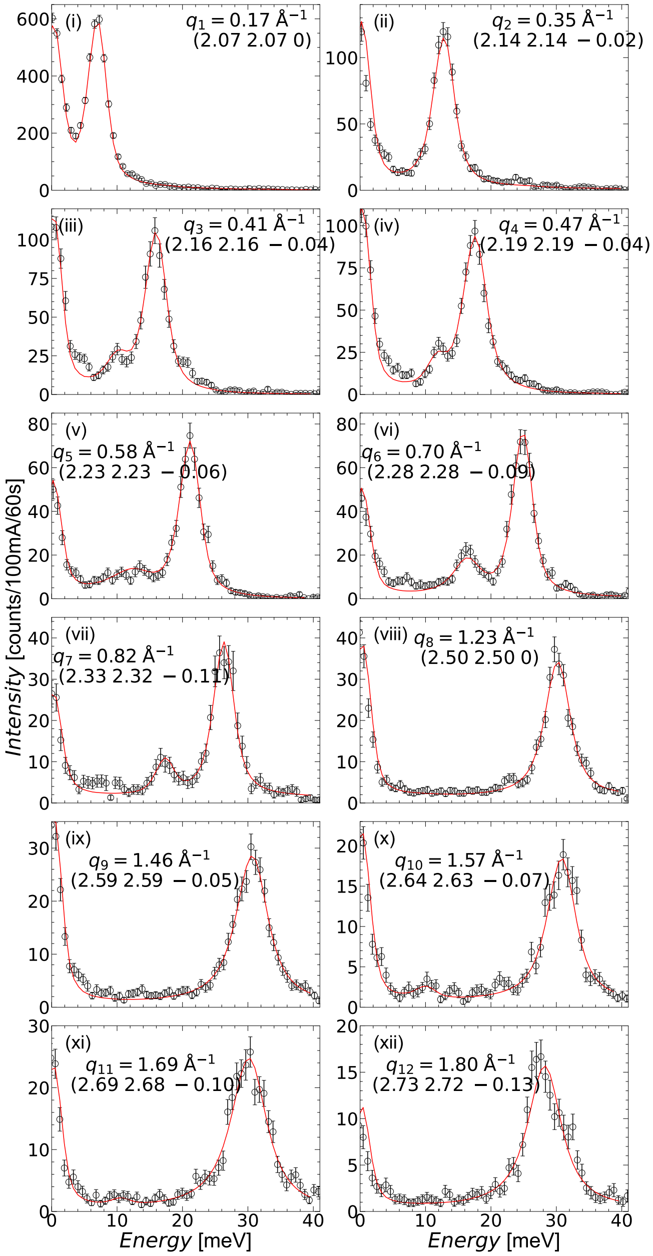

FIG. S21. Energy scans at 300 K of the longitudinal acoustic (LA) mode propagating along the [110] direction (LA_{110}), taken near the (220) Bragg peak. Experiments were made at the ID28@ESRF beamline, using the [999] reflection of the silicon monochromator.

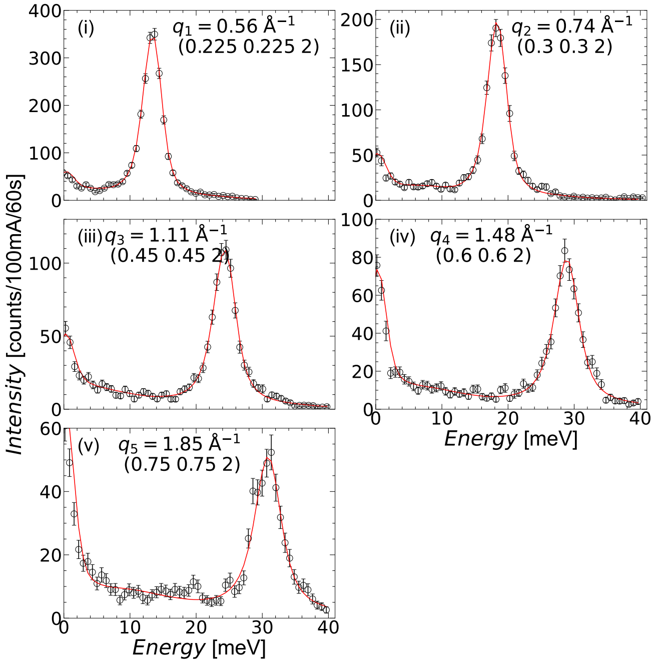

FIG. S22. Energy scans at 300 K of the transverse acoustic (TA) mode propagating along [110], polarized along [001] (TA_{110}^{001}), taken near the (002) Bragg peak. Experiments were made at the ID28@ESRF beamline, using the [999] reflection of the silicon monochromator.

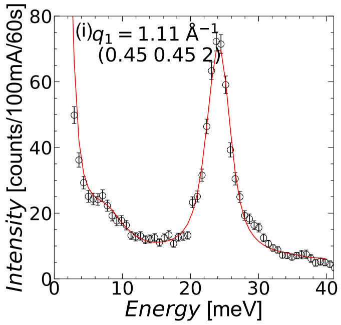

FIG. S23. Energy scan at 15 K of the transverse acoustic (TA) mode propagating along [110], polarized along [001] (TA_{110}^{001}), taken near the (002) Bragg peak. Experiment was made at the ID28@ESRF beamline, using the [999] reflection of the silicon monochromator.

SUPPLEMENTARY REFERENCES

[1] S. Pailhès, V. M. Giordano, P.-F. Lory, M. D. Boissieu, and H. Euchner, *Nanostructured Semiconductors*, edited by K. Termentzidis (Pan Stanford, 2017).

[2] A. Furrer, J. Mesot, and T. Strässle, *Neutron Scattering in Condensed Matter Physics* (WORLD SCIENTIFIC, 2009).

[3] G. L. Squires, *Introduction to the Theory of Thermal Neutron Scattering* (Cambridge University Press, 2012).

[4] M. d'Astuto and M. Krisch, High resolution inelastic x-ray scattering from thermal collective excitations, in *JDN 16 – Diffusion Inélastique des Neutrons pour l'Etude des Excitations dans la Matiére Condensée* (EDP Sciences, 2010).

[5] A. Q. Baron, High-resolution inelastic x-ray scattering i: Context, spectrometers, samples, and superconductors, in *Synchrotron Light Sources and Free-Electron Lasers* (Springer International Publishing, 2016) pp. 1643–1719.

[6] W. Reichardt, *MUPHOCOR, A Fortran Program To Determine the Phonon Density of States from Neutron Scattering Experiments* (1984).

[7] D. Richard, M. Ferrand, and G. J. Kearley, Analysis and visualisation of neutron-scattering data, Journal of Neutron Research **4**, 33 (1996).

[8] B. Hennion and P. Bourges, Afitv: Refinement program for triple axis spectrometer data.

[9] G. Shirane, S. M. Shapiro, and J. M. Tranquada, *Neutron Scattering with a Triple–Axis Spectrometer* (Cambridge University Press, 2015).

[10] M. Boudard, M. de Boissieu, S. Kycia, A. I. Goldman, B. Hennion, R. Bellissen, M. Quilichini, R. Currat, and C. Janot, Optic modes in the AlPdMn icosahedral phase, Journal of Physics: Condensed Matter **7**, 7299 (1995).

[11] P.-F. Lory, V. M. Giordano, P. Gille, H. Euchner, M. Mihalkovič, E. Pellegrini, M. Gonzalez, L.-P. Regnault, P. Bastie, H. Schober, S. Pailhes, M. R. Johnson, Y. Grin, and M. de Boissieu, Impact of structural complexity and disorder on lattice dynamics and thermal conductivity in the o-$Al_{13}Co_4$ phase, Phys. Rev. B **102**, 024303 (2020).

[12] M. de Boissieu, S. Francoual, M. Mihalkovič, K. Shibata, A. Q. R. Baron, Y. Sidis, T. Ishimasa, D. Wu, T. Lograsso, L.-P. Regnault, F. Gähler, S. Tsutsui, B. Hennion, P. Bastie, T. J. Sato, H. Takakura, R. Currat, and A.-P. Tsai, Lattice dynamics of the Zn-Mg-Sc icosahedral quasicrystal and its Zn-Sc periodic 1/1 approximant, Nat. Mater. **6**, 977 (2007).

[13] P.-F. Lory, *Dynamique de réseau et conductivité thermique dans les alliages métalliques complexes*, Ph.D. thesis, Université de Grenoble (2015).

[14] H. Euchner, S. Pailhès, L. T. K. Nguyen, W. Assmus, F. Ritter, A. Haghighirad, Y. Grin, S. Paschen, and M. de Boissieu, Phononic filter effect of rattling phonons in the thermoelectric clathrate $Ba_8Ge_{40+x}Ni_{6-x}$, Phys. Rev. B **86**, 224303 (2012).

[15] S. Pailhès, H. Euchner, V. Giordano, R. Debord, A. Assy, S. Gomès, A. Bosak, D. Machon, S. Paschen, and M. de Boissieu, Localization of propagative phonons in a perfectly crystalline solid, Phys. Rev. Lett. **113**, 025506 (2014).

[16] P.-F. Lory, S. Pailhès, V. M. Giordano, H. Euchner, H. D. Nguyen, R. Ramlau, H. Borrmann, M. Schmidt, M. Baitinger, M. Ikeda, P. Tomeš, M. Mihalkovič, C. Allio, M. R. Johnson, H. Schober, Y. Sidis, F. Bourdarot, L. P. Regnault, J. Ollivier, S. Paschen, Y. Grin, and M. de Boissieu, Direct measurement of individual phonon lifetimes in the clathrate compound $Ba_{7.81}Ge_{40.67}Au_{5.33}$, Nat. Commun. **8**, 491 (2017).

[17] H. Euchner, S. Pailhès, V. M. Giordano, and M. de Boissieu, Understanding lattice thermal conductivity in thermoelectric clathrates: A density functional theory study on binary Si-based type-I clathrates, Phys. Rev. B **97**, 014304 (2018).

[18] R. Viennois, M. M. Koza, R. Debord, P. Toulemonde, H. Mutka, and S. Pailhès, Anisotropic low-energy vibrational modes as an effect of cage geometry in the binary barium silicon clathrate $Ba_{24}Si_{100}$, Phys. Rev. B **101**, 224302 (2020).

[19] S. R. Turner, S. Pailhès, F. Bourdarot, J. Ollivier, S. Raymond, T. Keller, Y. Sidis, J.-P. Castellan, P.-F. Lory, H. Euchner, M. Baitinger, Y. Grin, H. Schober, M. de Boissieu, and V. M. Giordano, Impact of temperature and mode polarization on the acoustic phonon range in complex crystalline phases: A case study on intermetallic clathrates, Phys. Rev. Research **3**, 013021 (2021).

[20] M. S. Lucas, G. B. Wilks, L. Mauger, J. A. Muñoz, O. N. Senkov, E. Michel, J. Horwath, S. L. Semiatin, M. B. Stone, D. L. Abernathy, and E. Karapetrova, Absence of long-range chemical ordering in equimolar FeCoCrNi, Appl. Phys. Lett. **100**, 251907 (2012).

[21] M. S. Lucas, M. Kresch, R. Stevens, and B. Fultz, Phonon partial densities of states and entropies of Fe and Cr in bcc Fe-Cr from inelastic neutron scattering, Phys. Rev. B **77**, 184303 (2008).

[22] M. S. Lucas, L. Mauger, J. A. Muñoz, I. Halevy, J. Horwath, S. L. Semiatin, S. O. Leontsev, M. B. Stone, D. L. Abernathy, Y. Xiao, P. Chow, and

B. Fultz, Phonon densities of states of face-centered-cubic Ni-Fe alloys, J. Appl. Phys. **113**, 17A308 (2013)
